# Adjustable Intragastric Balloon Leads to Significant Improvement in Obesity-Related Lipidome and Fecal Microbiome Profiles: A Proof-of-Concept Study

**DOI:** 10.14309/ctg.0000000000000508

**Published:** 2022-06-07

**Authors:** Hisham Hussan, Barham K. Abu Dayyeh, Jun Chen, Stephen Johnson, Ken M. Riedl, Elizabeth M. Grainger, Jeffrey Brooks, Alice Hinton, Christina Simpson, Purna C. Kashyap

**Affiliations:** 1 Division of Gastroenterology, Hepatology, and Nutrition, The Ohio State University, Columbus, Ohio, USA;; 2 The Ohio State University Comprehensive Cancer Center, Columbus, Ohio, USA;; 3 Division of Gastroenterology, University of California Davis, Sacramento, CA, USA;; 4 Division of Gastroenterology and Hepatology, Mayo Clinic, Rochester, Minnesota, USA;; 5 Division of Biomedical Statistics and Informatics and Center for Individualized Medicine, Mayo Clinic, Rochester, Minnesota, USA;; 6 Division of Medical Oncology, The Ohio State University, Columbus, Ohio, USA;; 7 Spatz FGIA, Inc, Ft Lauderdale, Florida, USA;; 8 Division of Biostatistics, The Ohio State University, Columbus, Ohio, USA.

## Abstract

**INTRODUCTION:**

Intragastric balloons (IGBs) are a safe and effective treatment for obesity. However, limited knowledge exists on the underlying biological changes with IGB placement.

**METHODS:**

This single-institution study was part of an adjustable IGB randomized controlled trial. Subjects with obesity were randomized in a 2 is to 1 ratio to 32 weeks of IGB with diet/exercise counseling (n = 8) vs counseling alone (controls, n = 4). Diet/exercise counseling was continued for 24 weeks post-IGB removal to assess weight maintenance. We used mass spectrometry for nontargeted plasma lipidomics analysis and 16S rRNA sequencing to profile the fecal microbiome.

**RESULTS:**

Subjects with IGBs lost 15.5% of their body weight at 32 weeks vs 2.59% for controls (*P* < 0.05). Maintenance of a 10.5% weight loss occurred post-IGB explant. IGB placement, followed by weight maintenance, led to a −378.9 μM/L reduction in serum free fatty acids compared with pre-IGB (95% confidence interval: 612.9, −145.0). This reduction was mainly in saturated, mono, and omega-6 fatty acids when compared with pre-IGB. Polyunsaturated phosphatidylcholines also increased after IGB placement (difference of 27 μM/L; 95% confidence interval: 1.1, 52.8). Compared with controls, saturated and omega-6 free fatty acids (linoleic and arachidonic acids) were reduced after IGB placement. The fecal microbiota changed post-IGB placement and weight maintenance vs pre-IGB (*P* < 0.05). Further analysis showed a possible trend toward reduced Firmicutes and increased Bacteroidetes post-IGB and counseling, a change that was not conclusively different from counseling alone.

**DISCUSSION:**

IGB treatment is associated with an altered fecal microbiome profile and may have a better effect on obesity-related lipidome than counseling alone.

## INTRODUCTION

Obesity is projected to affect 1 in 2 US adults by 2030 (1). The resulting increase in obesity-related chronic conditions will likely put a significant strain on public health resources and the economy. Intragastric balloons (IGBs) were recently introduced to the United States as a practical weight loss intervention that improves many metabolic parameters observed with obesity (2). IGBs enable a significant weight loss of approximately 8–16 kg with 20%–30% excess weight loss after 6 months of therapy (3,4). As a result, IGBs may outperform other nonsurgical weight loss therapies, while having the benefit of being less invasive when compared with bariatric surgery. However, a paucity of data exists on the effect of IGBs on obesity-related comorbidities that are usually improved after bariatric surgery. In that regard, an improvement in lipid metabolism was shown to have prognostic implications and may mediate the beneficial effect of bariatric surgery on obesity-related conditions such as type-II diabetes mellitus and fatty liver disease (5–8). Thus, we aimed to investigate the effect of IGBs on lipid metabolism using serum lipidomics, a novel method for studying overall and individual biological lipid profiles, which is rapidly growing.

Limited knowledge also exists on the weight loss mechanisms of IGBs, which can improve outcomes and tailor IGB therapy. Some reports suggest IGB may cause weight loss by restricting gastric volume and providing a degree of delayed gastric emptying, thus leading to reduced caloric intake (9–11). Evolving literature also identifies a role for the gut microbiome in obesity, metabolic regulation of food intake, and success of weight loss. In support of this theory, it was suggested that fecal microbiota transplanted from bariatric subjects alters the metabolism and weight loss outcomes of germ-free mice (12–15). These microbiome alterations after bariatric surgery were linked to changes in intestinal fermentation and lipogenesis (14,16–18). In that context, evaluating the effect of IGBs on the microbiome can improve our knowledge of mechanisms responsible for inducing weight loss and altering lipid metabolism. Therefore, we explored whether IGB treatment is associated with a beneficial lipid profile and a significant change in the fecal microbiome. We examined these questions using the Spatz3 IGB—the only adjustable IGB that meets all the Tarpon Springs Comprehensive Workshop Standard for IGBs (19).

## METHODS

### Study flow and subjects

A total of 15 patients with IGBs and controls were recruited prospectively from Ohio State University as part of the Spatz3 randomized, controlled, multicenter study comparing the Spatz3 Adjustable Balloon System combined with diet and exercise with diet and exercise alone. The parent study evaluated the safety and effectiveness of the Spatz3 IGB in subjects aged 22 years and older with a body mass index (BMI) between 30 and 39.9 kg/m^2^ who had failed previous dietary and pharmaceutical weight loss programs (20). Our single-center study design is highlighted in Supplementary Figure 1, Supplementary Digital Content 1, http://links.lww.com/CTG/A829.

Per the parent study protocol, patients were randomized in a 2 to 1 ratio to 32 weeks of IGB with dietary/exercise counseling or dietary/exercise counseling alone (controls) (20). The experimental group received 24 weeks of counseling alone after 32 weeks. This extended follow-up was to assess weight maintenance after IGB explant, as was performed with other studies investigating the efficacy of IGBs (20,21). To prevent gastrointestinal bacterial overgrowth, the experimental group was placed on a probiotic containing 80% *Lactobacillus* sp. and 20% *Bifidobacterium* sp. during the 32-week IGB placement period, which was discontinued afterward. Subjects in both groups were counseled by a registered dietitian once a month and advised to consume a 1,000–1,200 kcal/d–deficit diet during their participation in the study. The physical activity counseling plan had 3 progressive stages, and our counseling plans are detailed elsewhere (20). Treatment arm subjects were evaluated at 18 ± 2 weeks, and those patients who did not reach goal weight and qualified based on pre-existing criteria had an adjustment procedure whereby the IGB volume was increased. Intolerant subjects were offered balloon volume-down adjustments to alleviate their intolerance.

### Exclusion criteria

The study was reviewed and approved by the OSU Institutional Review Board. Patients were excluded using exclusion criteria for the parent study (20). We excluded patients with any of the following characteristics: previous surgeries, motility disorders, structural anomalies, or inflammatory conditions of the gastrointestinal tract; insulin-dependent diabetes; chronic abdominal pain; hepatic insufficiency or cirrhosis; serious or uncontrolled psychiatric illness; alcoholism or drug addiction; patients who were unable or unwilling to take a prescribed proton pump inhibitor for the duration of the device implantation; pregnant or breastfeeding women; severe cardiopulmonary disease or other serious organic disease; subjects who had tested positive for *Helicobacter pylori* infection; current use of aspirin, anti-inflammatory agents, anticoagulants, corticosteroids, immunosuppressants, narcotics, diet pills, or drugs that affect the levels of serotonin in the body; eating disorders, genetic or hormonal causes for obesity, or participation in any clinical study, which could affect weight loss in the 6 months before study; and conditions that increase the risk of gastrointestinal bleeding.

### Outcomes

Our primary outcome was the effect of IGBs with diet and exercise counseling on the serum lipid concentrations and the fecal microbiome within subjects, compared with controls who underwent diet and exercise counseling alone. Furthermore, 7%–9% of patients with IGBs develop intolerance to the balloon, which requires early removal within the first weeks of placement (21,22). Therefore, as a secondary outcome, we looked at microbiome and lipidomics predictors of IGB intolerance leading to explant.

### Biospecimens and data collection

Stool and blood samples were collected in fasting subjects on the day of and before esophagogastroduodenoscopy conducted for IGB placement and IGB upward adjustment and on the day of IGB explant at 32 weeks. To assess a sustained effect, and compared with controls, fasting stool and blood samples were also collected after 24 weeks of counseling-alone intervention and from controls at the end of their 32 weeks of participation. Fecal samples were collected using a sterile, cotton-tip rectal swab (FLOQSwabs 552C, Copan, CA). Rectal swabs provide a quick method for microbial sampling (23,24). One swab was inserted into the anal canal, beyond the anal verge and rotated for 10 seconds before retrieval. Blood samples were collected using a standard blood collection kit. Swabs and blood samples were immediately placed on ice on retrieval and transported to be stored at −80 °C as per biorepository protocol. All samples were coded and blinded before testing. Clinical data, dietary patterns obtained by registered dietitians, and anthropometric data were collected by reviewing electronic medical records.

### Lipidomics sample preparation and analysis

Lipidomics testing was performed at OSU Comprehensive Cancer Center Nutrient and Phytochemical Analytics Shared Resource. Blood samples were drawn in polyester gel separators to allow separation of serum. Lipids were extracted by a Bligh and Dyer extraction and infused into a QTRAP 5500 mass spectrometer where lipid species are first separated by a Selexion differential ion mobility segment and further resolved by MS/MS transitions for each species. The infusion/MS platform is called the Lipidyzer (Sciex, Concord, Canada) and yields a comprehensive profile of plasma lipid similar to liquid chromatography-MS approach (25). Specifically, the Lipidyzer assay covers 13 different lipid classes and uses 54 stable isotope internal standards to provide semiquantitative results for as many as 1,100 lipid species. Lipid classes include phospholipids, ceramides, sphingolipids, free fatty acids, triacylglycerols, diacylglycerols, and cholesterol esters.

Changes in lipids across visits were assessed through the use of mixed linear models with random subject effects, and the Dunnett-Hsu method was used for post hoc comparisons for 32 weeks of IGB with or without 24 weeks of additional counseling to pre-IGB and controls. Furthermore, *t* tests were used to make comparisons between patients who had the IGB removed early vs those who completed the study. In this exploratory nontargeted lipidomics analysis, statistical significance was defined as a *P* value less than 0.05, as was conducted in previous studies (6,7,26). To reduce the number of the tests, we performed additional analyses only on lipid classes that seemed significantly different after IGB placement. SAS v9.4 (SAS Institute, Cary, NC) software was used to fit the models and perform the *t* tests.

### Microbiome sample preparation and analysis

DNA was extracted from stool samples using the Qiagen PowerSoil kit (Qiagen, Germantown, MD) and was quantified using a NanoDrop-8000 UV-Vis Spectrophotometer (Thermo Scientific, Wilmington, DE) and PicoGreen assays. Dual-index microbiome amplification of the V3–V5 region was conducted as previously described (27) with the primer sequences Meta_V3_F_Nextera TCGTCGGCAGCGTCAGATGTGTATAAGAGACAGCCTACGGGAGGCAGCAG and V5R_Nextera: GTCTCGTGGGCTCGGAGATGTGTATAAGAGACAGCCGTCAATTCMTTTRAGT.

Pooled samples were denatured with NaOH, diluted to 8 pm in Illumina's HT1 buffer, spiked with 10% PhiX, and heat denatured at 96 °C for 2 minutes immediately before loading. A MiSeq 600 cycle v3 kit was then used to sequence the sample (300 bp paired-end reads). Our microbiome sequencing and analytics methods are fully described in our supplemental material (Supplementary Digital Content 1, http://links.lww.com/CTG/A829).

## RESULTS

### Patient characteristics

Our study included 15 patients (93.3% female and 66.7% White), with a mean age of 42.5 ± 10.1 years. Patients had a mean BMI of 37.1 ± 2.4 kg/m^2^, and they used minimal medications at baseline. Eight of 11 patients with IGBs completed the 32 weeks of IGB placement and counseling, followed by 24 weeks of counseling alone. The remaining 3 patients with IGBs had to undergo early balloon removal within 3 weeks from IGB placement due to intolerance (intractable nausea and vomiting). At baseline, the controls (n = 4) had a similar BMI but were younger than the IGB and early removal groups (see Supplementary Table 1, Supplementary Digital Content 1, http://links.lww.com/CTG/A829 for demographic and medications data). Other than 1 male patient in the experimental group, all participants were females, and most of the participants were White. None of our recruited patients experienced diabetes before or during the whole study period. Patients with IGBs had a mean total weight loss percentage of 15.5% at 32 weeks as opposed to 2.59% for controls after 32 weeks of diet and exercise only (*P <* 0.05). Patients with IGBs maintained a 10.5% total weight loss percentage, 24 weeks from IGB explant.

### Changes in serum lipid composition within subjects undergoing IGB placement

**Figure 1.**
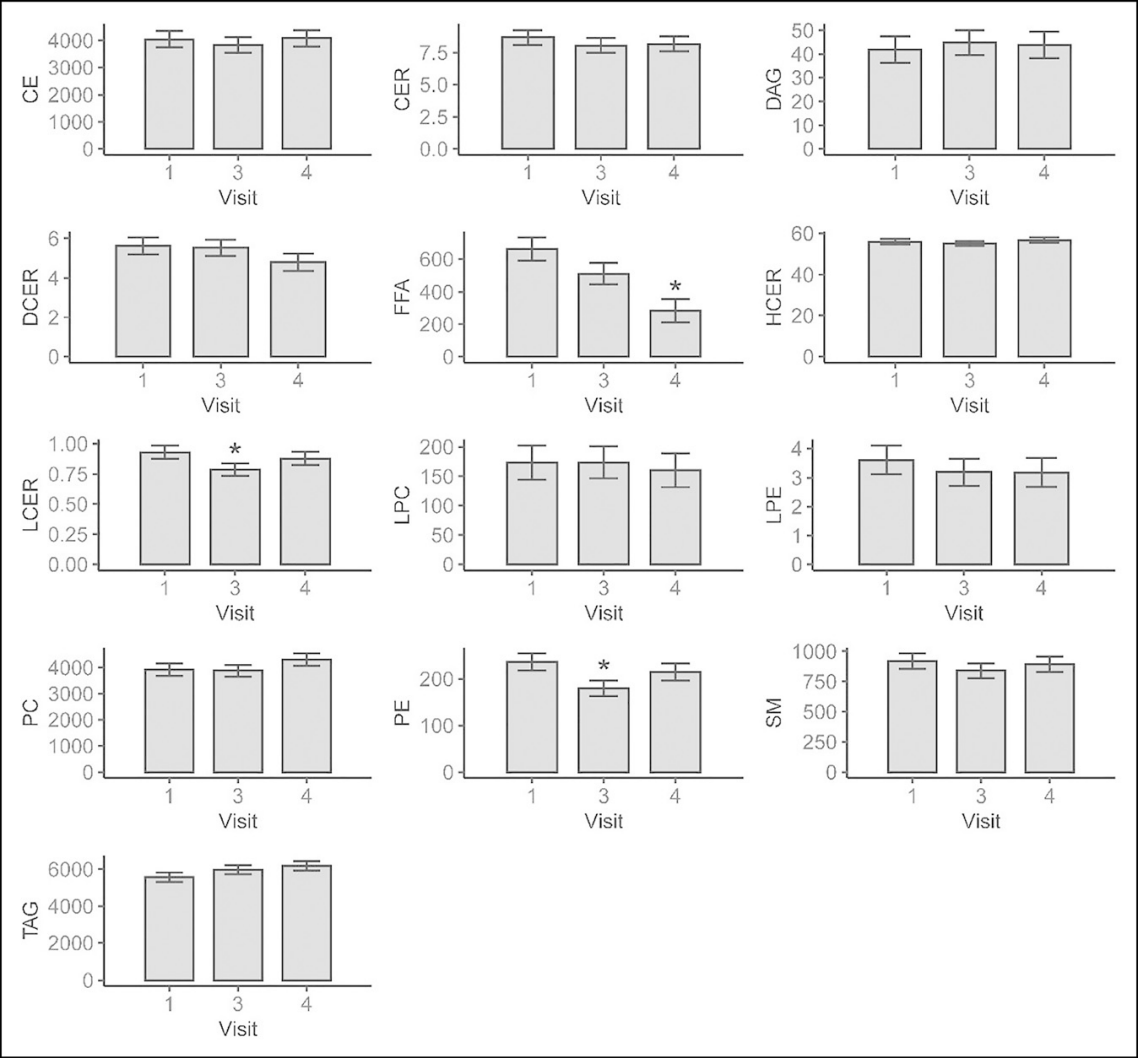
Least square means and standard errors from the models presented in Table 2 for each of the 13 total lipid sums. Asterisk indicate a statistically significant difference (*P* < 0.05) in visit 3 (after 32 weeks of IGB therapy) or visit 4 (after 32 weeks of IGB therapy and 24 weeks of weight maintenance) from visit 1 (Pre-IGB placement). Units are in micromole (μM) per liter. CE, cholesterol ester; CER, ceramide; DAG, diacylglyceride; DCER, dihydroceramide; FFA, free fatty acid; HCER, hexosylceramide; IGB, intragastric balloon; LCER, lactosylceramide; LPC, lysophosphatidylcholine; LPE, lysophosphatidylethanolamine; PC, phsphatidylcholine; PE, phosphatidylethanolamine; SM, sphingomyelins; TAG, triacylglyceridesPhosphatidylcholines.

Before IGB placement, the fatty acid classes with the highest concentrations were triacylglycerides (5,574.6 μM/L), cholesterol esters (4,050.9 μM/L), and phosphatidylcholines (3,927.1 μM/L). The remaining fatty acid classes had mean values reaching less than 1,000 μM/L, as summarized in Table 1. The differences between the levels of fatty acids at our time points are described in Table 2 and illustrated in Figure 1. Phosphatidylethanolamines, and to a lesser degree lactosylceramides, had a statistically significant reduction after 32 weeks of IGB treatment (difference of −56.5 μM/L; 95% confidence interval [CI]: −111.1, −1.9 and difference of −0.1 μM/L; 95% CI: 0.3, 0.0, respectively). Phosphatidylethanolamines and levels of lactosylceramides relapsed and were not different from their baseline values at the end of the weight maintenance period.

**Table 1.** The mean values and standard deviations for each of the 13 total lipid sums within subjects after IGB placement and weight maintenance (n = 7 patients)

	Pre-IGB placement		At IGB explant (32 wk)		After 24 wk of weight maintenance	
	Mean	SD	Mean	SD	Mean	SD
Triacylglycerides	5,574.6	501.1	5,973.5	434.8	6,188.2	970.3
Cholesterol esters	4,050.9	904.5	3,833.2	898.0	4,096.4	722.5
Phosphatidylcholines	3,927.0	771.5	3,882.0	641.2	4,315.5	505.8
Sphingomyelins	918.0	171.8	839.6	200.0	895.1	158.9
Free fatty acids	644.0	202.1	511.3	214.0	265.1	99.7
Phosphatidylethanolamines	236.3	38.8	179.3	53.8	214.4	50.2
Lysophosphatidylcholines	178.1	94.9	173.4	58.6	165.8	74.5
Hexosylceramides	55.9	2.3	55.0	2.7	56.7	4.7
Diacylglycerides	42.5	17.3	44.8	15.9	44.5	13.9
Ceramides	8.7	1.6	8.1	1.8	8.2	1.5
Dihydroceramides	5.6	1.4	5.5	1.1	4.8	0.9
Lysophosphatidylethanolamines	3.7	1.6	3.2	1.1	3.3	1.2
Lactosylceramides	0.9	0.2	0.8	0.1	0.9	0.9

The mean values are in micromole (μM) per liter.

IGB, Intragastric balloon.

**Table 2.** Results of mixed linear models fit for the significantly different lipid classes within 7 subjects who underwent IGB placement

	Difference between visit 3 and visit 1			Difference between visit 4 and visit 1		
	Diff.	95% CI	*P* value	Diff.	95% CI	*P* value
Free fatty acids	−150	(−378.2, 78.2)	0.1	−378.9	(−612.9, −145.0)	0.006
Lactosylceramides	−0.1	(−0.3, 0.0)	0.04	−0.1	(−0.2, 0.1)	0.5
Phosphatidylcholines	−34.1	(−468.0, 399.8)	0.9	388.4	(−48.4, 825.2)	0.08
Phosphatidylethanolamines	−56.5	(−111.1, −1.9)	0.04	−21.9	(−77.8, 34.1)	0.5

The Dunnett-Hsu adjustment method was used to make post hoc comparisons of visit 3 (after 32 wk of IGB therapy) or visit 4 (after 32 wk of IGB therapy and 24 wk of weight maintenance) to visit 1 (pre-IGB placement). Differences are in micromole (μM) per liter.

IGB, intragastric balloon.

Free fatty acids are the only lipid class to witness a reduction after IGB placement, which extended after IGB explant and weight maintenance (difference of −378.9 μM/L; 95% CI: −612.9, −145.0). On further analysis (Table 3), the largest drop in free fatty acids was in monosaturated (difference of −28 μM/L; 95% CI: −50.9, −5.0), followed by saturated (difference of −12.7 μM/L; 95% CI: −22.6, −2.7) and polyunsaturated fatty acids (difference of −7.1 μM/L; 95% CI: 213.0, −1.1). Among polyunsaturated free fatty acids, the levels of omega-6 fatty acids were more reduced when compared with omega-3 fatty acids. For example, the omega-3 fatty acids eicosapentaenoic acid and docosahexaenoic acid witnessed a minimal reduction after IGB placement and weight maintenance. Conversely, omega-6 fatty acids such as linoleic acid dropped significantly (difference of −70.9 μM/L; 95% CI: −117.4, −24.5), followed by arachidonic acid (difference of −2.5 μM/L; 95% CI: −4.2, −0.7). In parallel to a drop in free fatty acids, phosphatidylcholines tended to increase after IGB placement and weight maintenance (difference of 388.4 μM/L; 95% CI: 48.4, 825.2). On further stratification, phosphatidylcholines with polyunsaturated fatty acid moieties, especially omega-3 FA, were the only lipid form of phosphatidylcholines to significantly increase after IGB placement and weight maintenance (Table 3, difference of 27 μM/L; 95% CI: 1.1, 52.8).

**Table 3.** Results of mixed linear models fit for the individual fatty acid (FA) levels for free fatty acids and phosphatidylcholine within 7 subjects who underwent IGB placement

	Difference in fatty acids after 32 wk of IGB placement and 24 wk of weight maintenance compared with pre-IGB					
	Free fatty acids			Phosphatidylcholines		
	Difference	95% CI	*P* value	Difference	95% CI	*P* value
Saturated FA	−12.7	(−22.6, −2.7)	0.01	15.8	(−12.4, 43.9)	0.3
Monounsaturated FA	−28	(−50.9, −5.0)	0.01	22.2	(−35.4, 79.7)	0.5
Polyunsaturated FA	−7.1	(−13.0, −1.1)	0.02	27	(1.1, 52.8)	0.04
Omega-3 FA						
Linolenic acid (C18:3)	−4.9	(−8.8, −0.9)	0.01	103.6	(−8.79, 216.1)	0.07
Stearidonic acid (C18:4)	0	(−0.2, 0.1)	0.9	131.4	(−2.16, 265)	0.05
Eicosapentaenoic acid (C20:5)	−0.3	(−0.7, 0.1)	0.1	5.9	(−3.3, 15.2)	0.2
Docosapentaenoic acid (C22:5)	−0.5	(−1.0, 0.0)	0.06	3.5	(−7.7, 14.6)	0.6
Docosahexaenoic acid (C22:6)	−0.7	(−1.3, −0.1)	0.03	0.9	(−3.2, 5.1)	0.8
Omega-6 FA						
Linoleic acid (C18:2)	−70.9	(−117.4, −24.5)	0.007	103.6	(−8.8, 216.1)	0.07
Eicosadienoic acid (C20:2)	−0.7	(−1.2, −0.2)	0.009	0.6	(0.1, 1.2)	0.02
Arachidonic acid C20:4)	−2.5	(−4.2, −0.7)	0.008	12.3	(−56.2, 80.7)	0.8
Adrenic acid (C22:4)	−0.3	(−1.2, 0.7)	0.5	0.2	(−2.7, 3.0)	0.9
Omega-9 FA						
Mead acid (C20:3)	−1.0	(−1.7, −0.3)	0.009	6.1	(−31.4, 43.6)	0.8

The Dunnett-Hsu adjustment method was used to make post hoc comparison visit 4 (after 32 wk of IGB therapy and 24 wk of weight maintenance) from visit 1 (pre-IGB placement). Differences are in micromole (μM) per liter.

IGB, intragastric balloon.

### Changes in serum lipid profile after IGB placement compared with counseling alone

When compared with controls who underwent a lifestyle intervention alone, a reduction was observed with individual phosphatidylethanolamines with polyunsaturated and saturated FA at 32 weeks of IGB placement compared with controls (see Supplementary Table 2, Supplementary Digital Content 1, http://links.lww.com/CTG/A829). In addition, saturated free fatty acids and arachidonic free fatty acids decreased after IGB treatment and 24 weeks of weight maintenance compared with controls (difference of −6.7 μM/L; 95% CI: −13, −0.4 and difference of −1.3 μM/L; 95% CI: −2.5, −0.1, respectively, as summarized in Supplementary Table 3, Supplementary Digital Content 1, http://links.lww.com/CTG/A829). Phosphatidylcholines, on the contrary, did not change compared with controls who underwent the lifestyle intervention alone.

### Fecal microbiome changes within subjects with IGBs compared with those in controls

**Figure 2.**
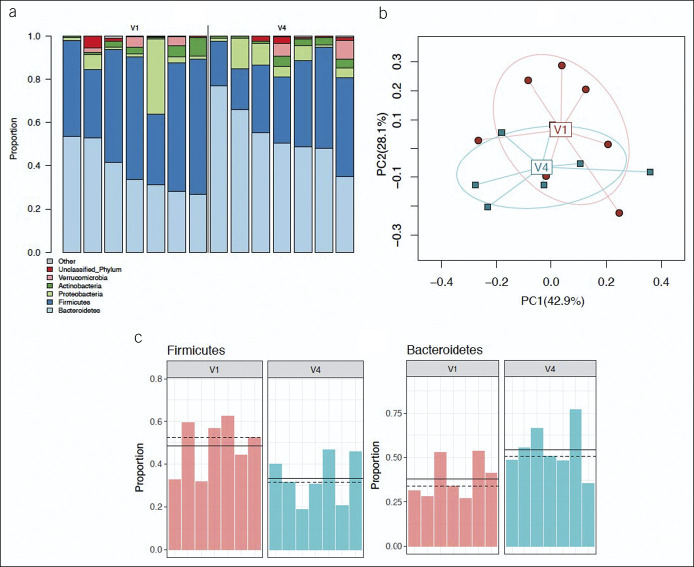
Fecal microbiome comparison within subjects between pre-IGB implantation (V1) and after 32 weeks of IGB treatment with diet and exercise followed, by 24 weeks of diet and exercise alone (V4). (**a**) Phylum-level profiles comparing V1 and V4; (**b**) PERMANOVA on weighted UniFrac *P* = 0.047 (all possible permutations), indicating potential different microbiota compositions in V4 vs V1; (**c**) differential taxa in V4 vs V1, FDR-corrected *P* value < 0.2 (permutation test): Reduced Firmicutes and increased Bacteroidetes. IGB, intragastric balloon.

The fecal microbiota composition and diversity changes pre-GB placement and post-IGB placement are shown in Supplementary Figure 2, Supplementary Digital Content 1, http://links.lww.com/CTG/A829. Firmicutes was the predominant phylum at baseline, followed by Bacteroidetes. There was no significant difference in fecal microbiota composition between baseline and 32 weeks of IGB treatment/counseling. However, an alteration in fecal microbiota composition was observed after 24 weeks of weight maintenance compared with pre-IGB placement (Figure 2a,b, weighted UniFrac, PERMANOVA, *P <* 0.05).

On further analysis, there was a potential trend toward decreased Firmicutes and increased Bacteroidetes after IGB treatment and weight maintenance compared with that at baseline (Figure 2c, q < 0.2). This microbiota composition after IGB placement and weight maintenance was not conclusively different from lifestyle intervention alone (see Supplementary Figure 3, Supplementary Digital Content 1, http://links.lww.com/CTG/A829).

We further investigated the change in *Fusobacterium* sp. after IGB placement because it was shown to increase after bariatric surgery (16). The mean relative abundance of Fusobacteria decreased by 43% with IGB placement, but this did not reach statistical significance, as shown in Supplementary Figure 4, Supplementary Digital Content 1, http://links.lww.com/CTG/A829.

### Microbiome and lipidomics predictors of early IGB removal

**Figure 3.**
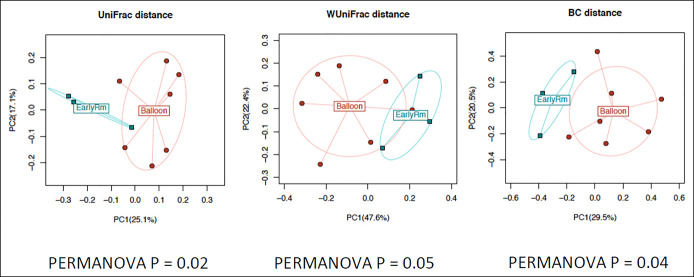
Pre-IGB implantation fecal microbiome composition of early IGB removal (blue) and completed IGB treatment (red) groups. No significant alpha-diversity associations, but significant beta-diversity associations. IGB, intragastric balloon.

The following subanalysis aimed to assess biomarker predictors of early IGB removal due to intolerance. There was a difference in baseline fecal microbiome structure and composition among those who later required early removal of IGB compared with patients who completed IGB treatment—as revealed by beta-diversity analysis (PERMANOVA, *P <* 0.05 for all investigated beta-diversity measures, see Figure 3). Further analysis revealed several differentially abundant bacterial taxa in the phyla Bacteroidetes, Fusobacteria, Firmicutes, and Actinobacteria.

At the genus level, genera such as *Porphyromonadaceae*, *Sneathia*, *Actinomyces*, and *Parvimonas* spp. were higher, whereas *Leptotrichia* was lower in the early removal group (see Supplementary Figure 5A, Supplementary Digital Content 1, http://links.lww.com/CTG/A829—significant families/genera, q < 0.1). At the OTU level, we identified an additional 10 differentially abundant OTUs (Supplementary Figure 5B—Supplementary Digital Content 1, http://links.lww.com/CTG/A829significant OTUs, q < 0.1). Detailed statistics of these differentially abundant taxa/OTUs are shown in Supplementary Table 7, Supplementary Digital Content 1, http://links.lww.com/CTG/A829.

Blood lipid analysis also identified a higher trend of lactosylceramide level in patients requiring early removal of IGB compared with those who completed treatment (*P* = 0.06). Further analysis revealed that saturated lactosylceramide was higher in the IGB than in the early removal group (difference of 0.02; 95% CI: 0.0, 0.04). One saturated PE (PE 16:0) also trended toward higher levels in the IGB compared with the early removal group (difference of 20.5; 95% CI −0.2, 41.3, *P* = 0.05).

## DISCUSSION

Obesity is associated with a high serum proportion of saturated and omega-6 fatty acids and low concentrations of omega-3 fatty acids (28–31). Our study is the first to show a sustained drop in saturated, monosaturated, and omega-6 polyunsaturated free fatty acid concentrations after IGB placement and weight maintenance. In parallel, polyunsaturated phosphatidylcholines increased after IGB/counseling treatment, which may provide a beneficial antioxidant protective effect (32). Compared with counseling, IGBs combined with counseling also decreased saturated and omega-6 free fatty acids. Indeed, free fatty acids are linked to adverse health outcomes, including nonalcoholic steatohepatitis and coronary artery disease (33,34). An improvement in serum free fatty acids is linked to reduced insulin resistance and better health outcomes (34). Therefore, our data suggest an amelioration of lipid profiles associated with obesity after IGB placement, which has more effect than counseling alone. In support of this theory, a recent study documented improvement in steatohepatitis after IGB therapy (35).

In our study, phosphatidylethanolamines and lactosylceramides were decreased during balloon removal and relapsed to baseline. Phosphatidylethanolamines during balloon removal were also decreased when compared with counseling alone. Phosphatidylethanolamines promote satiety and are produced from enterocytes in response to feeding (36). Previous evidence also suggests that lactosylceramides are associated with insulin resistance and fasting glucose and can be reduced with dietary weight loss interventions and bariatric surgery (7,37). Thus, our findings suggest these changes are due to the effect of balloon presence and associated dietary restriction. The normalization of these fatty acids after IGB removal could be due to the resumption of certain dietary patterns, which was evident by some weight regain at 24 weeks.

To our knowledge, this study is the first to demonstrate a lasting effect on the fecal microbiome, including a potential trend toward increased Bacteroidetes and decreased Firmicutes levels after IGBs and associated weight loss. Patients with IGBs who tolerated the treatment had a total weight loss percentage of 15.5% after IGB placement compared with less than 3% in controls. Patients with IGBs maintained a 10.5% TWL after 24 weeks from IGB removal. With this profound weight loss, IGB placement was associated with microbiome changes that are inconclusively different from nonsurgical weight loss seen in our controls and previous studies (38,39). Notably, the experimental group received the probiotic during the 32 weeks of IGB placement. The probiotic was composed of 80% *Lactobacillus*, a Firmicute. Therefore, the probiotic can theoretically negate a possible decrease in Firmicutes from weight loss during IGB presence. This may explain why there was no difference in the microbiome at 32 weeks compared with pre-IGB placement. The experimental group did not receive any probiotics for the 24 weeks after IGB removal, and probiotics are less likely to colonize the mucosa after cessation of probiotics (40). Therefore, the probiotics given during the first 32 weeks should not affect the microbiome comparisons at 24 weeks after IGB removal. This may explain our significant differences at 24 weeks after IGB explant compared with pre-IGB.

In contrast to our findings, Roux-en-Y gastric bypass leads to a distinct microbiome signature with decreased Firmicutes and increased *Bacteroides* sp., Fusobacteria, and *Escherichia coli* (16). Vertical sleeve gastrectomy also leads to an increased Firmicutes to Bacteroidetes ratio (18,41). The different microbiome profiles seen with bariatric surgery compared with IGB may indicate the selective effect of surgical alterations on the colonic microbiome instead of the profound weight loss seen with surgery.

The other novel hypothesis that we tested was predictors of early balloon removal. Interestingly, there was a significant difference in baseline fecal microbiome composition (beta diversity; UniFrac and Bray Curtis) among those who later required early removal of IGB and patients who completed treatment. These microbiome differences may suggest an underlying altered gastrointestinal function, or alternatively, dietary habits predisposing patients to more severe IGB symptoms. Consistent with microbiome changes, there was an increase in certain saturated fatty acids (phosphatidylethanolamines and lactosylceramides). Certainly, as opposed to unsaturated fatty acids, saturated fatty acids can induce inflammation that may alter gastrointestinal motility (42–44). Thus, altered gastric motility may have led to the severe symptoms with IGB placement, thus leading to early removal.

Our study is the first to examine microbiome and lipidomics changes after IGB placement. The strength of our methods was the recruitment of subjects that passed the strict inclusion and exclusion criteria required for the parent FDA-regulated study (20). Subjects were also randomized to the intervention vs the control, which adds rigor to the study.

However, a limitation of the study is the small sample size, which does not provide the statistical power to detect moderate changes or relations between the microbiome and weight loss outcomes. The sample size is also too small for us to assess confounders such as age, sex, or race on microbiome changes after IGB placement when comparing the experimental group with controls or adults with early balloon removal. We could not investigate direct relations between the microbiome and lipid profiles after IGB placement due to our limited sample size. Nevertheless, we could still detect long-term changes in the microbiome and lipidomics patterns suggesting hypotheses that warrant testing in more extensive studies. Furthermore, these differences would not affect the comparisons within subjects' post-vs pre-IGB placement. None of our recruited patients had diabetes or were on metformin during enrollment or during the whole study period. Thus, our study can only be applicable to adults without diabetes.

In conclusion, this study shows a change in microbiome and lipidomics with IGB placement, suggesting possible mechanisms for weight loss and the beneficial effect of IGBs on obesity-related comorbidities. In parallel, our pilot data explored the utility of the microbiome and lipids in treatment stratification by facilitating identification of patients most likely to tolerate IGBs placement, thus avoiding an expensive procedure in those likely to fail. These data provide preliminary data for larger trials to verify our findings and to determine whether the microbiome changes influence the change in lipidome to improve the beneficial effects of IGBs.

## CONFLICTS OF INTEREST

**Guarantor of the article:** Hisham Hussan, MD.

**Specific author contributions:** H.H. was involved in the study conception, sample collection, design, interpretation of data, manuscript drafting, and critical revision. A.H., J.C., and S.J. analyzed the data, and provided a critical revision of the manuscript. The above authors had full access to all the data in the study and take responsibility for the data's integrity and the data analysis' accuracy. C.S. and E.G. were involved in recruiting patients, managing informed consent, biospecimens transport and storage, and critical review of manuscript. K.M.R. performed the lipidomics testing at NPASR and reviewed the manuscript. S.C., B.A., J.B., and K.P. were involved in the design, data interpretation, and critical revision of the manuscript. All gave final approval of the submitted manuscript and take responsibility for the integrity of the work. Finally, we acknowledge Dr. Steven K. Clinton for his support with providing insight and laboratory support for the analysis.

**Financial support:** This work was supported by the NIH R01 grant DK114007 (PCK), Mayo Clinic Center for Individualized Medicine.

**Potential competing interests:** B.K.A.: Boston Scientific (consultant), BFKW (consultant), USGI (consultant, grant/research support), Endogenex (consultant), Endo-TAGSS (consultant), Metamodix (consultant), Olympus (speaker), Medtronic (speaker, grant/research support), Johnson & Johnson (speaker), EndoGastric Solutions (speaker and research support), Apollo Endosurgery (research support), Cairn Diagnostics (grant/research support), Aspire (grant/research support), and Spatz (research support). P.C.K.: Member of the advisory board of Novome, and consults for Pendulum Therapeutics and Otsuka Pharmaceutical. J.B.: Chief Operating Officer and shareholder of Spatz FGIA, Inc. Other authors have no conflicts of interests.

**IRB approval statement:** The study was reviewed and approved by the Ohio State University Institutional Review Board.

**Study Highlights**

**WHAT IS KNOWN**

✓ Improvements in the obesity-related lipidome mediate the beneficial effect of weight loss.
✓ The effect of intragastric balloon (IGB) placement on serum lipid profiles is unknown.
✓ In addition, limited data exist on microbiome changes with balloon placement.

**WHAT IS NEW HERE**

✓ IGBs are associated with improvement in the obesity-related lipidome, mainly decreased saturated mono and omega-6 free fatty acids.
✓ The improvement in the obesity-related lipidome with IGBs and lifestyle counseling is more effective than counseling alone.
✓ Despite a more pronounced weight loss, the fecal microbiome after IGBs and counseling is not conclusively different from counseling alone.

## Supplementary Material
