# Supplementary material for: Adjustable Intragastric Balloon Leads to Significant Improvement in Obesity-Related Lipidome and Fecal Microbiome Profiles: A Proof-of-Concept Study
Source: Clin Transl Gastroenterol. 2022 Jun 7;13(7):e00508. doi: 10.14309/ctg.0000000000000508 (PMC10476793; doi:10.14309/ctg.0000000000000508)
Supplement: Supplementary file 1 [file ct9-13-e00508-s001.docx]

**Microbiome sequencing and analytic methods**

Paired-end sequence reads were processed via the *hybrid-denovo* bioinformatics pipeline[^27^](#_ENREF_27), which clustered these paired-end reads into operational taxonomic units (OTUs) at the 97% similarity level. OTUs were assigned taxonomy using the RDP classifier trained on the GreenGenes database (v13.5)[^28^](#_ENREF_28). Singleton OTUs as well as samples with less than 2,000 reads were removed as a quality control (QC) step. Microbial α-diversity and β-diversity were analyzed based on the rarefied OTU data. Microbial α-diversity reflects species richness and evenness within the microbial populations, and three representative α-diversity measures (observed number of OTUs, Shannon index, and Inverse Simpson index) were investigated. Microbial β-diversity (between-sample diversity)—reflecting the shared diversity between bacterial populations and different β-diversity measures—provides distinctive views of the community structure and composition. Three representative β-diversity measures (unweighted and weighted UniFrac plus Bray-Curtis distance) were calculated (R package “GUniFrac” and “vegan”)[^29^](#_ENREF_29). OTU data were also summarized into different taxonomic ranks based on their associated taxonomy.

To test the association between the covariate and α-diversity, a linear model or linear mixed effects model (with α-diversity being the outcome) was used for independent and dependent (i.e., paired) data, respectively. To test the association between the covariate and β-diversity, we used PERMANOVA, a distance-based analysis of variance based on permutation ( “adonis” function in the R “vegan” package)[^30^](#_ENREF_30). Permutation was performed within subjects for paired data. Ordination plots were generated using principal coordinate analysis (PCoA) (“cmdscale” function in the R “vegan” package) for visualization of the overall microbiome structure/composition based on β-diversity. Differential abundance analysis was performed at the phylum, class, order, family, genus, and OTU levels—and taxa with prevalence less than 10% or with a maximum proportion less than 0.2% were excluded from testing to reduce the number of the tests.

The count data were normalized into relative abundances by dividing by the GMPR size factor, and was further square-root transformed[^31^](#_ENREF_31). To identify differentially abundant taxa while accounting for the non-normality of the abundance data, a permutation-based approach based on the F-statistics of a linear model (transformed relative abundance as the outcome variable) was performed, and within-subject residual permutation was used to account for within-subject correlations for paired data[^32^](#_ENREF_32). False discovery rate (FDR) control (B-H procedure, ‘p.adjust’ in standard R packages) was used to correct for multiple testing of the permutation-based tests for each taxonomic rank[^33^](#_ENREF_33) and FDR-adjusted p-values or q-values < 0.1 were considered significant.

**Supplemental Figure 1:** Study flow and samples collection time-points.

**
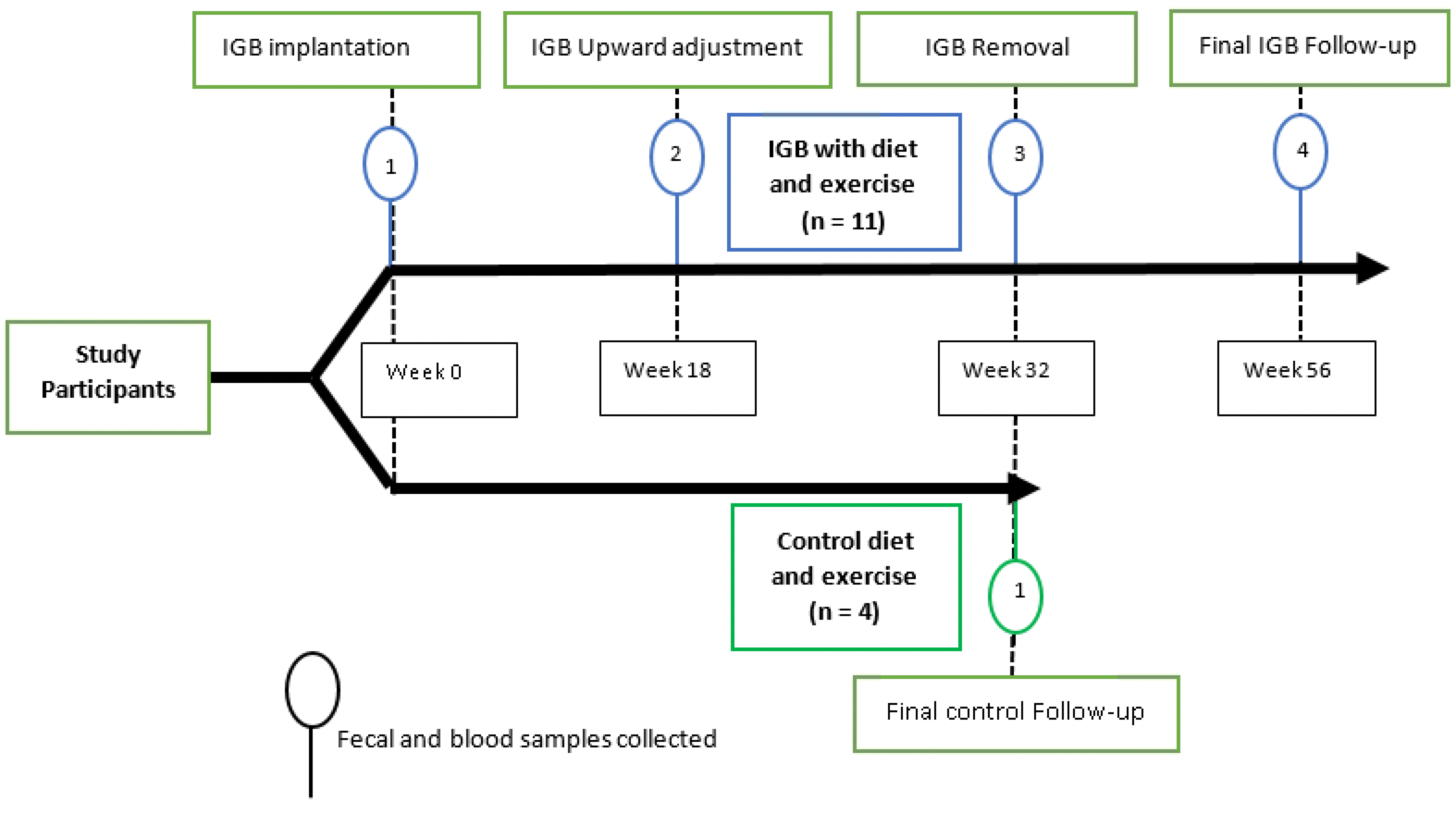
**

**Supplemental Table 1**. Summary of demographics and other characteristics of the sample population.

|  | Control arm  (n=4) | | IGB arm  (n=8) | | Early IGB Removal  (n=3) | |
| --- | --- | --- | --- | --- | --- | --- |
| Demographics | *n* | *%* | *n* | *%* | *n* | *%* |
| Age (mean, SD) | 36.8 | 7.9 | 45.4 | 9.4 | 42.3 | 14.8 |
| Sex |  |  |  |  |  |  |
| Male | 0 | 0.00 | 1 | 12.5 | 0 | 0.0 |
| Female | 4 | 100.0 | 7 | 87.5 | 3 | 100.0 |
| Race |  |  |  |  |  |  |
| White | 2 | 50.0 | 7 | 87.5 | 1 | 33.3 |
| African American | 2 | 50.0 | 1 | 12.5 | 2 | 66.7 |
| Smoking | 0 | 0.0 | 0 | 0.0 | 1 | 33.3 |
| Medication Prior to Balloon Placement | | | | | | |
| PPI | 0 | 0.0 | 1 | 12.5 | 1 | 33.3 |
| Probiotics | 0 | 0.0 | 0 | 0.0 | 1 | 33.3 |
| MVI | 1 | 25.0 | 3 | 50.0 | 2 | 66.7 |
| Statin | 0 | 0.0 | 1 | 12.5 | 0 | 0.0 |
| Fish oil | 0 | 0.0 | 1 | 12.5 | 1 | 33.3 |
| Weight History | *mean* | *SD* | *mean* | *SD* | *mean* | *SD* |
| Baseline BMI | 37.9 | 1.5 | 36.8 | 2.8 | 36.6 | 3.2 |
| % TWL | -2.6 | 5.1 | -10.5 | 7.1 | -- | -- |
| BMI Change | -1 | 1 | -3.5 | 2.3 | -- | -- |

**Supplemental Table 2**. Comparison of the individual fatty acid (FA) levels for **Phosphatidylethanolamine** between the 7 IGB patients at 32 weeks of IGB with lifestyle counseling to 4 control patients at 32 of counseling alone. P-values are from t-tests. Means and differences are in micromole (uM) per litter

|  | Controls  (n=4) | | IGB  (n=8) | | IGB - Control | |  |
| --- | --- | --- | --- | --- | --- | --- | --- |
|  | *Mean* | *SD* | *Mean* | *SD* | *Difference* | *95% CI* | *p-value* |
| Phosphatidylethanolamine (PE) saturated FA | | | | | | | |
| Saturated FA | 53.1 | 10.9 | 37.3 | 10.7 | -15.8 | (-30.5, -1.1) | 0.03 |
| Mono-unsaturated FA | 15.8 | 5.1 | 9.5 | 2.8 | -6.3 | (-11.2, -1.4) | 0.01 |
| Poly-unsaturated PE FA | 24.7 | 4 | 16.3 | 5.2 | -8.4 | (-15.1, -1.8) | 0.01 |
| Omega-3 FA | | | | | | | |
| Linolenic acid (C18:3) | 9.3 | 4.4 | 6.2 | 2.5 | -3.1 | (-7.44, 1.3) | 0.1 |
| Stearidonic acid  (C18:4) | 2 | 1.2 | 0.9 | 0.4 | -1 | (-2.03, -0.0) | 0.04 |
| Eicosapentaenoic acid (C20:5) | 30.1 | 10.1 | 22.4 | 7.9 | -7.7 | (-19.5, 4) | 0.17 |
| [Docosapentaenoic acid](https://en.wikipedia.org/wiki/Docosapentaenoic_acid) (C22:5) | 46.1 | 8.2 | 28.4 | 8 | -17.7 | (-28.7, -6.7) | 0.005 |
| Docosahexaenoic acid (C22:6) | 47.4 | 10.4 | 37 | 10.4 | -10.4 | (-24.5, 3.7) | 0.1 |
| Omega-6 FA | | | | | | | |
| Linoleic acid (C18:2) | 30 | 12 | 17.9 | 6.9 | -12.1 | (-24.0, -0.2) | 0.04 |
| Eicosadienoic acid (C20:2) | 0.4 | 0.1 | 0.3 | 0.1 | -0.2 | (-0.3, 0.0) | 0.04 |
| Arachidonic acid C20:4) | 74.8 | 17.3 | 45.8 | 17.5 | -29 | (-52.7, -5.3) | 0.02 |
| Docosadienoic acid (C22:2) | 0 | 1 | 0 | 0 | 0 | (-0.02, 0.01) | 0.1 |
| Adrenic acid (C22:4) | 6.4 | 1.4 | 3.3 | 1.1 | -3.1 | (-4.7, -1.5) | 0.002 |
| Omega-9 FA | | | | | | | |
| Mead acid (C20:3) | 8.3 | 3 | 4.3 | 1.5 | -4.1 | (-6.9, -1.3) | 0.009 |

^1^ Standard deviation not provided when there is only a single observation

**Supplemental Table 3**. Comparison of the individual fatty acid (FA) levels for **free fatty acids** between the 7 IGB patients at 32 weeks of IGB with lifestyle counseling to 4 control patients at 32 of counseling alone. P-values are from t-tests. Means and differences are in micromole (uM) per litter

|  | Controls Visit 3  (n=4) | | IGB Visit 4  (n=7) | | IGB Visit 4 – Controls Visit 3 | | |  |
| --- | --- | --- | --- | --- | --- | --- | --- | --- |
|  | *Mean* | *SD* | *Mean* | *SD* | *Difference* | *95% CI* | | *p-value* |
| Saturated FA | 18.5 | 6.1 | 11.8 | 3.3 | -6.7 | (-13, -0.4) | | 0.03 |
| Mono-unsaturated FA | 40.4 | 12.3 | 28.6 | 18.4 | -11.9 | (-35.4, 11.7) | | 0.2 |
| Poly-unsaturated FA | 11.7 | 3.5 | 9.2 | 3.8 | -2.5 | (-7.8, 2.8) | | 0.3 |
| Omega-3 FA | | | | | | |  | |
| Linolenic acid (C18:3) | 6.5 | 2.6 | 4.8 | 1.9 | -1.7 | (-4.8, 1.4) | | 0.2 |
| Stearidonic acid  (C18:4) | 0.3 | --^1^ | 0.4 | 0.1 | 0.1 | (-0.8, 1) | | 0.4 |
| Eicosapentaenoic acid (C20:5) | 0.8 | 0.2 | 0.7 | 0.2 | 0 | (-0.3, 0.3) | | 0.7 |
| [Docosapentaenoic acid](https://en.wikipedia.org/wiki/Docosapentaenoic_acid) (C22:5) | 1.3 | 0.2 | 1.1 | 0.3 | -0.2 | (-0.7, 0.3) | | 0.2 |
| Docosahexaenoic acid (C22:6) | 1.3 | 0.1 | 1.1 | 0.5 | -0.3 | (-1.2, 0.7) | | 0.5 |
| Omega-6 FA | | | | | | | | |
| Linoleic acid (C18:2) | 74.1 | 21.2 | 53.4 | 21.6 | -20.6 | (-51.1, 9.8) | | 0.1 |
| Eicosadienoic acid (C20:2) | 1.5 | 0.4 | 1.2 | 0.2 | -0.3 | (-0.7, 0.1) | | 0.1 |
| Arachidonic acid C20:4) | 4.4 | 1 | 3.1 | 0.8 | -1.3 | (-2.5, -0.1) | | 0.03 |
| Docosadienoic acid (C22:2) | 0.5 | 0.1 | -- | -- | --^1^ | --^1^ | | --^1^ |
| Adrenic acid (C22:4) | 1 | 0 | 0.7 | -- | -0.3 | (-0.5, -0.1) | | 0.04 |
| Omega-9 FA | | | | | | | | |
| FFA(FA20:3) | 2.1 | 0.7 | 1.6 | 0.4 | -0.5 | (-1.2, 0.2) | | 0.1 |

|  |  |  |  |  |  |  |  |  |  |  |
| --- | --- | --- | --- | --- | --- | --- | --- | --- | --- | --- |

^1^ Standard deviation not provided when there is only a single observation

**Supplemental Figure 2:** Microbiome changes during the course of IGB placement and removal. Sequence depth to philum (A) and genus level (B). Visits are: (V1) Pre-IGB implantation; (V2) IGB volume increase at 8-12 weeks after implantation; (V3) At time of IGB removal (36 weeks from implantation); (V4) 24 weeks from IGB removal.

**
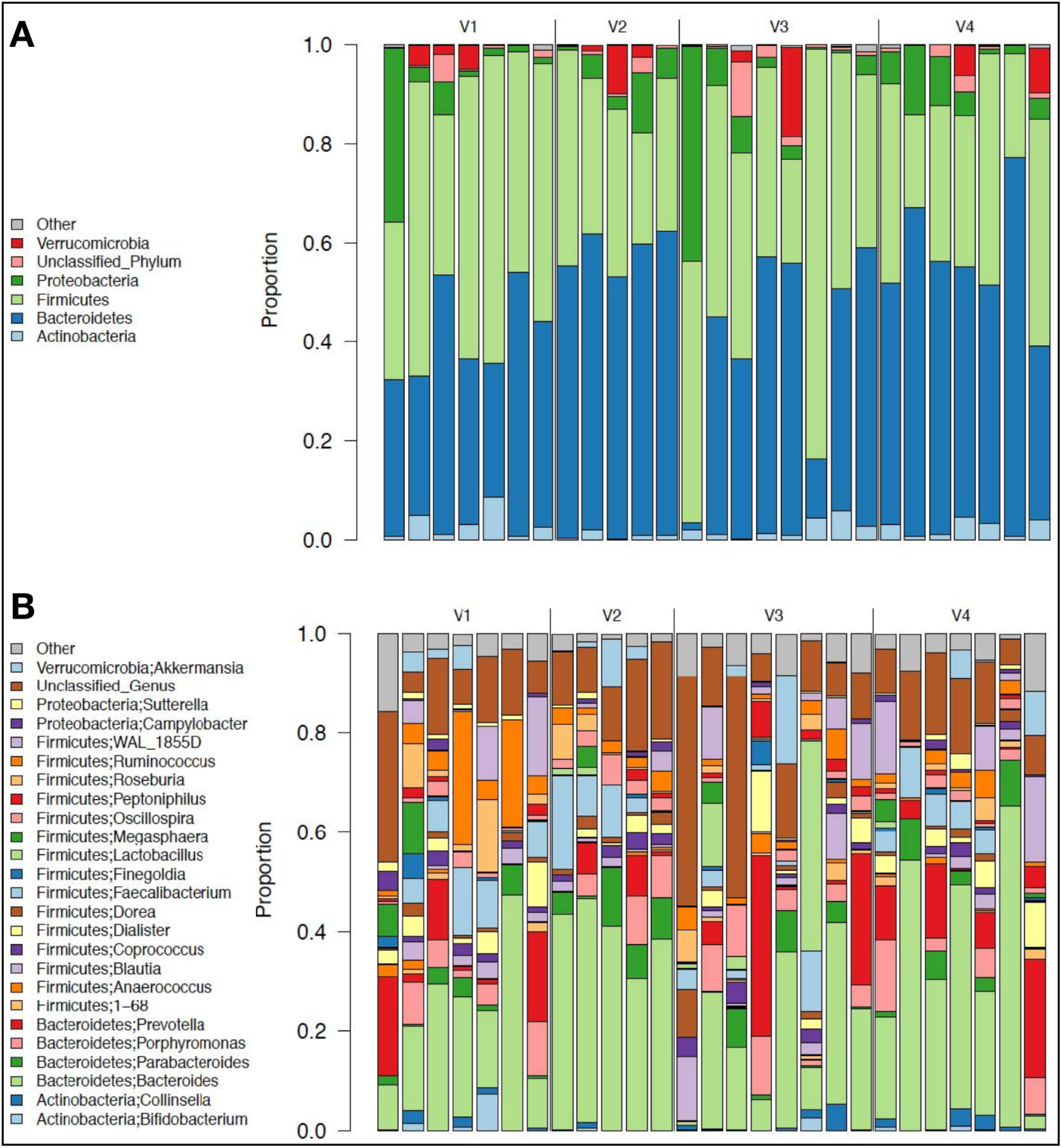
**

**Supplemental Figure 3:** Microbiome composition at after IGB and weight maintenance compared to controls who underwent diet and exercise only. Association p-values for all α-diversity and β-diversity measures were greater than 0.2.


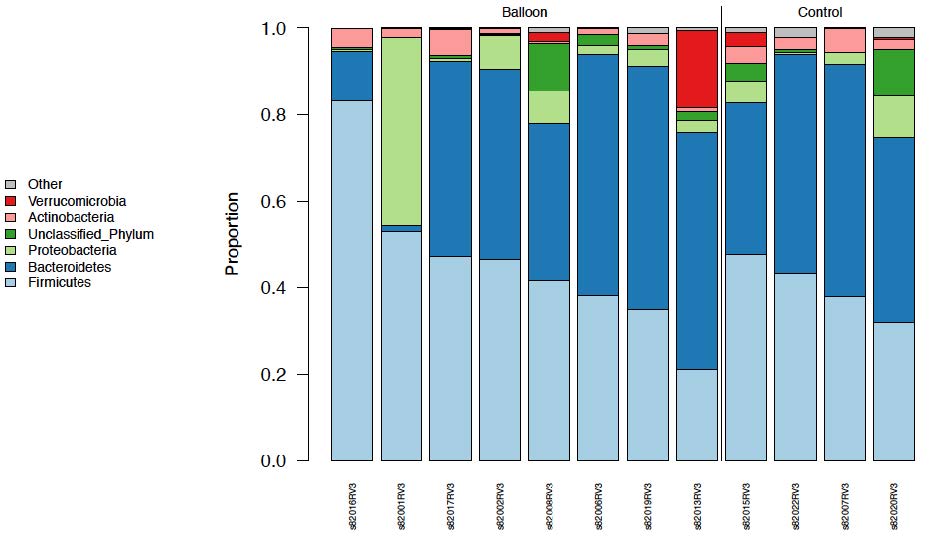


**Supplemental Figure 4:** Changes in *Fusobacterium* pre- and post- IGB placement.

**
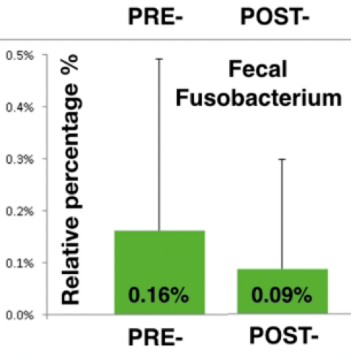
**

**
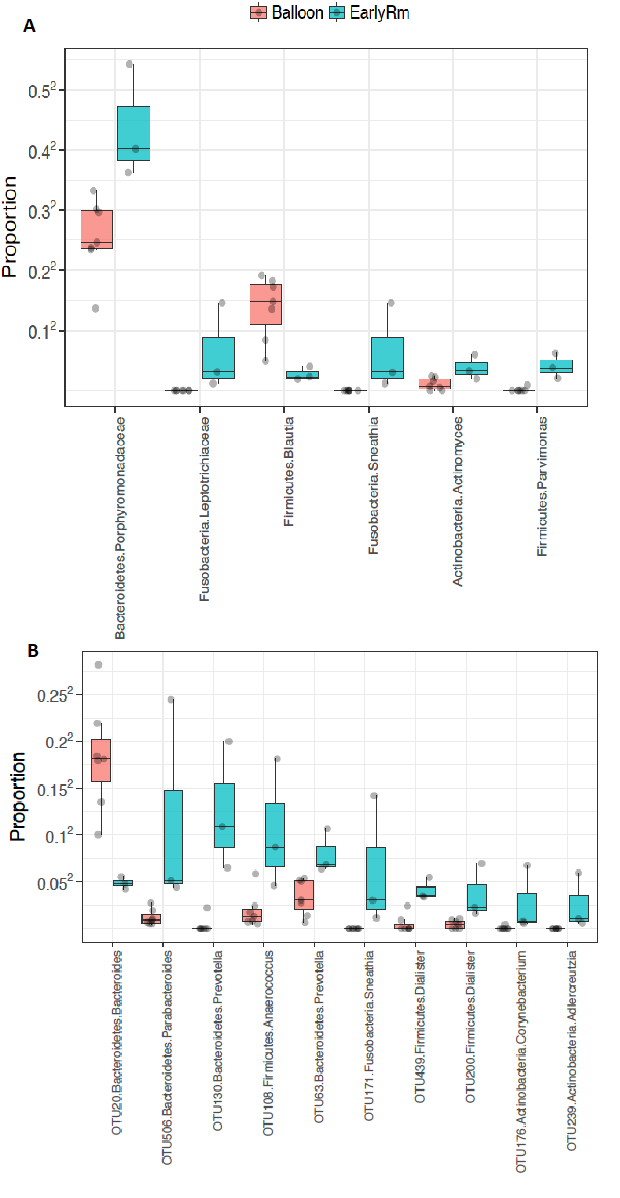
Supplemental Figure 5**. Pre-IGB relative differences of fecal microbial taxa between early IGB removal (blue) and completed IGB treatment (red) groups: (A) Differential taxa (Phylum to Genus level); (B) Differential OTUs. FDR-corrected p-value < 0.1

**Supplemental Table 7**. Raw p-value, FDR-adjused p-value (q value), and the average abundance in patients who completed IGB treatment and those that had early removal of IGB, as well as log abundance ratio ****
